# Three-Dimensionally Printed Agarose Micromold Supports Scaffold-Free Mouse Ex Vivo Follicle Growth, Ovulation, and Luteinization

**DOI:** 10.3390/bioengineering11070719

**Published:** 2024-07-15

**Authors:** Emily J. Zaniker, Prianka H. Hashim, Samuel Gauthier, James A. Ankrum, Hannes Campo, Francesca E. Duncan

**Affiliations:** 1 Department of Obstetrics and Gynecology, Feinberg School of Medicine, Northwestern University, Chicago, IL 60611, USA; emily.zaniker@northwestern.edu (E.J.Z.); prianka.hashim@northwestern.edu (P.H.H.); samuelj.gauthier@gmail.com (S.G.); 2 Roy J. Carver Department of Biomedical Engineering, Pappajohn Biomedical Institute, University of Iowa, Iowa City, IA 52245, USA; james-ankrum@uiowa.edu

**Keywords:** reproductive biology, engineered microenvironment, bioengineering, 3D printing, ovulation, follicle culture, ovary, scaffolds, agarose

## Abstract

Ex vivo follicle growth is an essential tool, enabling interrogation of folliculogenesis, ovulation, and luteinization. Though significant advancements have been made, existing follicle culture strategies can be technically challenging and laborious. In this study, we advanced the field through development of a custom agarose micromold, which enables scaffold-free follicle culture. We established an accessible and economical manufacturing method using 3D printing and silicone molding that generates biocompatible hydrogel molds without the risk of cytotoxicity from leachates. Each mold supports simultaneous culture of multiple multilayer secondary follicles in a single focal plane, allowing for constant timelapse monitoring and automated analysis. Mouse follicles cultured using this novel system exhibit significantly improved growth and ovulation outcomes with comparable survival, oocyte maturation, and hormone production profiles as established three-dimensional encapsulated in vitro follicle growth (eIVFG) systems. Additionally, follicles recapitulated aspects of in vivo ovulation physiology with respect to their architecture and spatial polarization, which has not been observed in eIVFG systems. This system offers simplicity, scalability, integration with morphokinetic analyses of follicle growth and ovulation, and compatibility with existing microphysiological platforms. This culture strategy has implications for fundamental follicle biology, fertility preservation strategies, reproductive toxicology, and contraceptive drug discovery.

## 1. Introduction

Follicles, which consist of oocytes surrounded by somatic support cells, known as granulosa cells, are the fundamental functional units of the ovary [1,2]. The process of follicle growth and development, known as folliculogenesis, is a tightly coordinated process that depends on bi-directional communication between the oocyte and granulosa cells and, ultimately, leads to the development of a high-quality gamete capable of being fertilized [3,4,5,6]. During early folliculogenesis, up until the secondary follicle stage, the somatic cells initially proliferate independent of signaling from pituitary gonadotropins and support the growth and development of the oocyte through this bi-directional communication [7,8,9,10]. In a gonadotropin-dependent phase, preantral follicles mature to the antral stage, which is defined by formation of a fluid-filled antral cavity. At the time of ovulation, a surge of luteinizing hormone (LH) triggers a series of remodeling events within the antral follicle wall that culminates in follicular rupture and release of a fertilization-competent gamete [11,12,13,14,15,16,17]. The egg, surrounded by specialized granulosa cells called cumulus cells, then transits to the fallopian tube or oviduct for possible fertilization [11,18,19,20,21]. Following ovulation, the remnants of the follicle wall involute to form a corpus luteum, a transient endocrine organ that produces the progesterone necessary to sustain early pregnancy [14,15,16].

The ability to recapitulate and study folliculogenesis, ovulation, and luteinization ex vivo has many potential research and clinical applications for reproductive biology and health. The development of improved ex vivo models that recapitulate key features of follicle development provide a tightly controlled model system to study follicle-intrinsic processes independent of the ovarian microenvironment. Additionally, ex vivo follicle growth and ovulation assays can be utilized for reproductive toxicology studies, the development of non-hormonal contraceptives, and drug screening to determine impacts on folliculogenesis and hormone production [22,23,24,25,26]. These techniques also have potential in clinical fertility preservation [27,28,29,30,31] or species conservation [32,33].

Given the broad basic, translational, and clinical utility of being able to grow follicles outside the context of the ovary, numerous ex vivo follicle growth strategies have been developed over the past several decades [34]. The simplest follicle culture techniques are classified as two-dimensional, whereby isolated follicles are cultured directly on a plastic dish, a plate coated with various ECM components, or on a membrane insert [34,35,36]. Though these culture systems are technically simple and have high throughput, they only support short-term cultures and are characterized by loss of follicle integrity [34,35,36]. Development of three-dimensional (3D) culture strategies that use hydrogels to encapsulate follicles have been instrumental in better modeling follicle growth and development in a way that better recapitulates in vivo physiology, including maintenance of follicle structure, improved meiotic maturation rate, and increased capacity for steroid hormone production [34]. Moreover, these systems support the sustained culture of follicles from large mammalian species, including nonhuman primates and humans [37,38,39,40,41,42,43]. Alginate, an inert biomaterial that is naturally derived from algae, is utilized to create hydrogels at varying concentrations to support follicle growth due to a pore size that allows diffusion of essential molecules from the surrounding media [26,34,35,36,37,38,44,45,46,47,48,49,50,51,52,53,54,55,56,57,58]. Alginate-based assays also recapitulate key transcriptomic signatures of follicle growth and ovulation in a mouse model [58]. Modifications to the 3D culture strategy have further improved follicle development and gamete quality outcomes, including modification of the materials used in cultures, such as fibrin-alginate networks or tunable polyethylene glycol (PEG) hydrogels. Co-cultures with a variety of somatic cells, extracellular matrix proteins (ECM), scaffolds printed with biopolymers including collagen, or fibers with ECM-sequestering peptides, and the addition of different supplements in the growth media, have also been used to improve the biomimetic aspect of cultures and follicle viability [1,44,45,46,47,48,49,50,51,52,53,54,55,56,57,58,59]. 

Despite their overall success, existing 3D culture protocols for multilayer secondary follicles have some key disadvantages. The first is that these methods are technically challenging and laborious, as individual isolated follicles must be encapsulated manually [54]. It is also not possible to automatically image these follicles as the free-floating beads do not maintain a consistent orientation over the culture period. In addition, for ovulation and oocyte maturation studies, the hydrogel must be completely removed [54]. Although engineering the tunability of hydrogels with biomaterials is an advantage of 3D cultures, lot-to-lot variability of components may be introduced across experiments and limit reproducibility.

Thus, the goal of this study was to broaden the accessibility of advanced follicle culture methods by engineering a custom scaffold-free agarose micromold with microwells designed based on the dimensions of antral-stage follicles. We established the simultaneous culture of 10 multilayer secondary follicles per mold, which itself is placed into an individual well of a standard 24-well plate. Using this novel system to culture mouse follicles, we demonstrated improved follicle growth and ovulation outcomes compared to the standard alginate-based encapsulated follicle culture method and comparable or improved follicle survival, oocyte maturation, and luteinization outcomes. In addition, follicles cultured in this system established and exhibited a spatial polarization, wherein follicle rupture and cumulus–oocyte complex (COC) release at the time of ovulation occurred from a consistent apical orientation, suggesting that this system may better mimic additional aspects of in vivo ovulation physiology. Lastly, we easily and automatically quantified follicle growth and ovulation kinetics with timelapse imaging.

Overall, this novel scaffold-free strategy for follicle culture offers technical simplicity, ease of culturing and tracking multiple follicles simultaneously, and maintenance of the complex architecture of follicular morphology and gamete meiotic competence observed in existing 3D culture systems. Lastly, the micromolds are compatible with a tissue-agnostic microphysiological device [59], which allows complex multi-tissue modeling of follicular development and ovulation under dynamic culture conditions. Thus, this method of mammalian follicle culture is likely more accessible and can be widely adopted for various studies with direct research and clinical relevance.

## 2. Materials and Methods

### 2.1. Animals

Follicles were collected from ovaries of CD-1 mice between postnatal days (PND) 14 and 16. CD-1 pups were purchased at PND5 with foster dams and housed in the animal facilities of Northwestern University prior to use (Envigo, Indianapolis, IN, USA). Mice were kept in barrier facilities under temperature, humidity, and light control (14 h light/10 h dark cycles) with food and water provided ad libitum. Animal procedures were approved by the Institutional Animal Care and Use Committee (IACUC) of Northwestern University.

### 2.2. Three-Dimensional Design and Generation of Agarose Micromolds

Using the dimensions of the developing secondary follicle and the surface area of the LATTICE culture wells, a computer-aided design (CAD) of the mold was made in Fusion360 (Autodesk Inc., San Rafael, CA, USA). The mold was designed to measure 15.6 mm long to allow for placement in a standard 24-well culture plate with a press-fit shape to prevent movement. The design included 25 microwells with 500 by 700 by 700 µm dimensions to fit a terminal-size antral stage follicle with extra space to prevent compression or growth restriction. Then, a manufacturing method to cast agarose molds was devised. Here, an initial 3D print was performed using a Form 2 SLA printer using Clear v4 resin (Formlabs Inc., Somerville, MA, USA) and postprocessed following the manufacturer’s instructions. The culture wells of the print were sprayed extensively with isopropyl alcohol during the wash steps and dried with compressed air before heat curing.

This mold was then completely casted in 6-well plates using Ecoflex™ 00-45 (Smooth-On Inc., Macungie, PA, USA) following the manufacturer’s instructions and allowed to cure for minimum of 4 h in a vacuum. The resulting silicone mold was then removed and treated with a soap-based mold release mixture, allowed to dry, and casted again with Ecoflex™ 00-45 for 4 h, generating the final mold to be used for the agarose mold. The silicone molds were rinsed with ethanol and autoclaved before each use. To cast the agarose micromold, approximately 600 µL of molten sterile 1.5% agarose (Hoefer, Holliston, MA, USA, Cat. No. GR140-500) solution in Dulbecco’s Phosphate Buffered Saline without calcium or magnesium (DPBS-/-, Thermo Fisher Scientific, Waltham, MA, USA) was pipetted into the silicone mold and allowed to solidify. The agarose molds were placed in 24-well plates and stored for up to 2 weeks in sterile DPBS or equilibrated with culture media (incubated twice for at least 1 h in 0.75 mL of media) to prepare for ex vivo follicle growth.

### 2.3. Ex Vivo Follicle Growth

To isolate follicles, we used a brief enzymatic digestion. Ovaries were cut into quarters before being incubated in L15 media (Thermo Fisher Scientific, Waltham, MA, USA) supplemented with 1% penicillin-streptomycin (Thermo Fisher Scientific), 25 μg/mL Liberase (Sigma-Aldrich, St. Louis, MO, USA), and 200 μg/mL DNase I (Qiagen, Hilden, Germany) for 20 min at 37 °C on an orbital shaker [30,33,35,39,47]. Enzymes were quenched with 10% fetal bovine serum (FBS; Peak Serum Inc., Wellington, CO, USA) prior to manual dispersion of digested ovary pieces to release follicles. Multilayer secondary follicles were selected based on size (150–180 μm diameter) and morphology of >2 layers of granulosa cells.

Follicles were then separated into two groups. The first group was encapsulated and cultured in 0.5% (*w*/*v*) alginate hydrogel (Sigma-Aldrich, Cat. No. 71238) as previously described [54]. Briefly, individual multilayer secondary follicles were placed in alginate solution and then beads were formed and cross-linked using a solution of 50 mM CaCl2 and 140 mM NaCl (Thermo Fisher Scientific) for 2 min. The second group of follicles were seeded in individual microwells of agarose micromolds that were equilibrated as described above with 10 follicles in each mold. Individual alginate beads were cultured in individual wells of a 96-well plate while groups of 10 follicles in agarose micromolds were cultured in individual wells of a 24-well plate. To have a similar culture medium to follicle ratio all experiments were performed using 10 follicles in 750 μL of growth media within the molds or one follicle per 100 μL of growth media in the control condition. The growth media contained 50% αMEM Glutamax (Thermo Fisher Scientific) and 50% F-12 Glutamax (Thermo Fisher Scientific) supplemented with 3 mg/mL bovine serum albumin (BSA; Sigma-Aldrich), 10 mIU/mL recombinant follicle stimulating hormone (rFSH; Gonal-F, Merck & Co., Rahway, NJ, USA), 1 mg/mL bovine fetuin (Sigma-Aldrich), and 5 μg/mL insulin–transferrin–selenium (ITS, Sigma-Aldrich). The surrounding empty wells were filled with sterile DPBS-/- (Thermo Fisher Scientific) to maintain humidity and prevent evaporation during culture. Follicles were cultured in a humidified environment of 5% CO_2_ in air at 37 °C for a total of 8 days with half of the growth media replaced every 48 h. Media was collected on day 8 of follicle culture for downstream hormone analysis. Follicles were imaged every other day of culture using the EVOS cell imaging system (Thermo Fisher Scientific) at 10× magnification. Follicle survival was assessed by identifying unhealthy and dying follicles based on morphology (opaque or dark color, granular appearance, and misshapen or extruded oocyte) or stagnant size from day 4 to 8 of culture. Average follicle diameter was measured in surviving follicles using ImageJ.

### 2.4. Ex Vivo Ovulation and Luteinization Assay

After 8 days of culture, follicles that had reached the antral stage, as evidenced by a terminal diameter of >350 μm and a visible fluid-filled antral cavity, were selected for ovulation induction. In the alginate-encapsulated group, follicles were first removed from the alginate beads by incubating them at 37 °C for 20 min in L15 media with 1% FBS and 10 IU/mL alginate lyase from flavobacterium multivorum (Sigma-Aldrich) and then washed in in L15 media supplemented with 0.5% penicillin–streptomycin and 1% FBS. Individual follicles removed from the alginate beads were then transferred to an equilibrated in vitro maturation (IVM) media in a 96-well plate. The IVM media consisted of 50% αMEM Glutamax and 50% F-12 Glutamax supplemented with 10% FBS, 1.5 IU/mL human chorionic gonadotropin (hCG; Sigma-Aldrich), 10 ng/mL epidermal growth factor (EGF; BD Biosciences, Franklin Lakes, NJ, USA), and 10 mIU/mL rFSH (Merck & Co.). For follicles grown in the agarose micromolds, the growth media was aspirated and replaced with 750 μL of warmed IVM media. Once transferred to the IVM media, all follicles were cultured in a humidified environment of 5% CO_2_ at 37 °C for 14–16 h to induce ovulation and oocyte maturation. After ovulation, cumulus–oocyte complexes were collected for imaging with EVOS system at 10× magnification and whole-mount immunocytochemistry. After follicle rupture, the residual follicle wall involutes and undergoes luteinization to form a progesterone-producing corpus luteum. This process also occurs in ex vivo and can be monitored by culturing the residual follicle wall after ovulation for an additional 48 h in media containing 50% αMEM Glutamax (Thermo Fisher Scientific) and 50% F-12 Glutamax (Thermo Fisher Scientific) with 10%FBS (Peak Serum Inc.). Media was collected on day 8, after induction of ovulation, and after 48 h of post-ovulatory culture for downstream hormone analyses. Ex vivo corpora lutea were then fixed in 3.8% Paraformaldehyde (PFA) (Thermo Fisher Scientific) with 0.1% Triton X-100 (Alfa Aesar, Haverhill, MA, USA) to allow for permeabilization at 37 °C for 1 h for histology.

### 2.5. Timelapse Culture and Analysis

Timelapse cultures were placed in an incubator containing an RK-10A mount holding a Dino-Lite Edge AF4915ZTL microscope with N3C-R light focuser (Dino-Lite Digital microscopes, Torrance, CA, USA). The plates and microscope were oriented to optimally focus on 10 follicles in the capture field at approximately 75× magnification. Images were taken throughout the culture every 30 min during the follicular and luteal phases or every 10 min for ovulation. Images were then analyzed using ImageJ version 1.54f. Firstly, images for each culture phase (follicular, ovulation, and luteal) were opened as a virtual stack and aligned using the scale invariant feature transform (SIFT) plugin. Then, the background was subtracted, stacks were tilted 10 degrees if necessary, and converted to 8-bit greyscale. This was then converted to a black and white mask using intermodes thresholding. The surface area, circularity, and other morphological metrics of each follicle were measured using particle analysis. Ovulation was visualized further by generating a kymograph; for each follicle, a segmented line was selected through the direction of ovulation and resliced. Cultures were imaged on the EVOS cell imaging system every 2 days to compare timelapse surface area measurements with standard average diameter measurements.

### 2.6. Whole-Mount Immunocytochemistry and Spindle Analysis

Eggs that had extruded a polar body following ovulation induction and IVM of cultured follicles were isolated from COCs, and whole-mount immunocytochemistry (ICC) was performed to assess spindle morphology as a marker of gamete quality. Cumulus cells were manually stripped from the eggs, which were then fixed in 3.8% PFA with 0.1% Triton X-100 for 20 min at 37 °C. Eggs were then washed three times in blocking buffer, which contained PBS with 10% Tween-20 (Sigma-Aldrich), 20% NaN3 (Honeywell, Charlotte, NC, USA), and 0.3% BSA. Oocytes were then permeabilized using PBS (Thermo Fisher Scientific) with 0.1% Triton X-100, 20% NaN3, and 0.3% BSA for 15 min at room temperature and then incubated with Alexa Fluor 488-conjugated anti-alpha-tubulin antibody (Sigma-Aldrich, Cat. No. T6074) at a concentration of 1 μg/mL for 2 h, protected from light with gentle rocking. Oocytes were then washed three times with blocking buffer prior to mounting on microscope slides with Vectashield Antifade Mounting Medium containing 4′,6-diamindo-2-phenylindole (DAPI; Vector Laboratories, Newark, CA, USA). Follicles were then imaged with a Leica TCS Sp5 confocal microscope using a 63× objective (Leica Microsystems, Wetzlar, Germany). Each oocyte was imaged with optical z-sections at 1.0 µm intervals throughout the entire MII spindle. Spindles were analyzed in ImageJ by creating a 3D projection, rotating spindles to be aligned with the focal plane, and measuring the length, width, and area of the MII spindles.

### 2.7. Progesterone Quantification Immunoassay

The concentration of progesterone (P4) was measured in conditioned media from day 8 of follicle culture, media collected after ovulation, and media collected 48 h later using the P4 ELISA Kit (Cayman Chemical, Ann Arbor, MI, USA) according to manufacturer’s instructions with at least three biological replicates. Media from alginate-encapsulated follicles was pooled across 10 wells.

### 2.8. Histologic Analysis

To analyze follicle and corpus luteum morphology, we completed histologic analysis of cultured follicles. To increase the efficiency of follicle histology and allow for sectioning of multiple follicles within the same plane, we developed a technique to utilize the agarose micromolds as a scaffold. The wells of agarose micromolds were filled with 0.5% (*w*/*v*) alginate hydrogel (Sigma-Aldrich), which served as a substrate to hold follicles in place during tissue processing and embedding. Fixed follicles and corpora lutea were washed three times in 0.5% alginate before transferring to alginate-filled microwells in an array of up to 10 ex vivo corpora lutea. The entire micromold filled with alginate was submerged in a solution of 50 mM CaCl2 and 140 mM NaCl (Thermo Fisher Scientific) for 4 min before inverting and crosslinking for an additional 2 min. After crosslinking, the alginate with embedded fixed tissue was carefully removed from the agarose micromolds using forceps and placed in a solution of Alcian Blue dye (Vector Laboratories, Newark, CA, USA) for no more than 15 s until the alginate was stained light blue to assist in visualization during embedding and sectioning. The alginate was then serially dehydrated by washing with 50%, 60%, and 70% ethanol each for 15 min. The encapsulated tissues were then processed using an automated tissue processor (Leica Biosystems, Buffalo Grove, IL, USA) according to standard processing protocols and embedded in paraffin wax. The tissues were then serially sectioned at intervals of 5 μm, placed on slides, and stained with hematoxylin and eosin (H&E) to assess follicle morphology and luteinization. Luteinization was assessed by measuring the cytoplasmic-to-nuclear ratio in ImageJ using the Trainable Weka Segmentation plugin.

### 2.9. Statistical Analysis

All statistical analyses were performed using GraphPad Prism 10.0 (GraphPad Software Inc., San Diego, CA, USA). All data in figures are either represented as the mean ± standard deviation or as a percentage. For analysis of follicle growth, hormone production, and nuclear to cytoplasmic ratio, a two-way ANOVA test with multiple comparisons was used to determine significant differences between groups. Ovulation and oocyte maturation rates were compared using a chi-squared test. Timelapse growth curves were fitted using four parameter logistic regression models, from which steepness of curve and time to 50% of top growth plateau were calculated. In all comparisons, a *p*-value of <0.05 was classified as statistically significant.

## 3. Results

### 3.1. Rationale Design and Precision Engineering of Agarose Micromolds

The aim of this study was to design a culture array with specialized microwells for murine ovarian follicles and compare its functionality to an established eIVFG alginate culture method [34,54]. The mold adhered to several design criteria. Firstly, the engineered microenvironment had to accommodate the significant size increase of mouse late-secondary follicles with a starting diameter of ~150–180 µm to preovulatory follicles, which typically reach a terminal diameter of ~350–450 µm [6,54,60]. Secondly, all follicles in the mold had to be cultured in the same focal plane, which also increased experimental throughput and enabled timelapse imaging. The overall structure of the mold also had to protect the follicles from movements between different types of culture wells and during media changes to simplify the seeding procedure and assure continuous imaging. Further, the mold had to be immobilized in both 24-well and LATTICE culture plates using a press-fit to enable automatic imaging and future microfluidic experiments [59]. Lastly, to improve adoption and usability, the mold had to be made using accessible, biocompatible, and sterilizable materials and with standard manufacturing methods.

**Figure 1:**
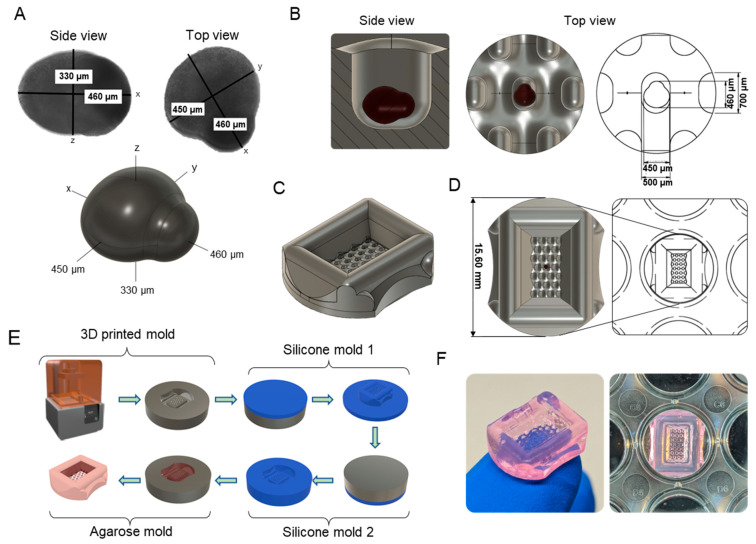
Design criteria towards designing a scaffold-free mold for follicle culture. (**A**) Firstly, a 3D model was made, informed by the dimensions of a large preovulatory follicle from standard eIVFG cultures. (**B**) The 700-by-500-micron microwells allow for follicle growth and ovulation, while remaining confined within defined dimensions. (**C**) The mold design includes a central cavity with 25 microwells. (**D**) The outer dimensions are the same as those of a culture well in the LATTICE system or a 24-well plate. (**E**) A negative master mold was 3D printed, followed by two silicone molding steps. The final functional mold is used to create the agarose micromolds. (**F**) The mold reproduces the intended design features, and firmly fits into culture well.

To determine the optimal dimensions of the microwells, measurements of fully grown preovulatory follicles were derived from established eIVFG models [34,54]. A computer-aided design (CAD) measuring 330 by 450 by 460 µm was used to represent the follicle during the design phase (Figure 1A). To not impede growth while still maintaining follicles in defined positions, 500 by 700 µm microwells were selected (Figure 1A,B). Once these dimensions were chosen, the rest of the mold was built around it; 25 microwells were distributed in regular intervals in a central cavity within the culture well (Figure 1C). Moreover, the mold measured 15.6 mm long, which is also the diameter used in a standard 24-well culture plate. This design resulted in a press-fit that prevented movement during culture (Figure 1D).

Once a CAD was made that met all design criteria, the appropriate materials and manufacturing methods were selected. Agarose was determined to be the best candidate for this study because it is affordable, inert, and commonly found in biological research labs. Agarose is a versatile biomaterial with excellent biocompatibility and well-defined mechanical properties that do not change substantially during short-term culture. Moreover, agarose allows sufficient oxygen and nutrient transport for cell growth [61,62]. Agarose is also used to reproducibly create custom ultra-low adhesion microwells for organoid culture [63]. Agarose has been used to culture and image embryos [64,65], as well as culture ovarian fragments [66], follicles [67], and oocytes [68], thereby demonstrating safety and utility for sensitive reproductive cells and tissues.

To micropattern and cast agarose, a first mold with the desired design was printed using stereolithography (SLA) 3D printing, which is a cost-effective method that allows for high customizability and fast design iterations with less specialized equipment [63,69,70]. This “vat photopolymerization” method was selected over direct printing of agarose-based bio-inks because of its ubiquity, ease-of-use, and excellent printing resolution. SLA 3D printing accomplishes this by curing a liquid resin layer-by-layer with a light source. This makes printing orientation an important consideration because it can affect build stability, precision, and optical clarity of the print [70]. Printing the same design with a 45-degree tilt along the x or y axis leads to distortions due to the 25-micron deposition (Figure S1). Resin-based 3D printed designs have been incorporated directly with ex vivo culture systems [63,70]. However, concerns regarding their cytotoxicity require extensive washes or post-processing to improve biocompatibility [71,72]. The direct use of these 3D prints for the ex vivo culture of reproductive tissues containing germ cells would likely have unfavorable outcomes due to this cytotoxicity [72,73]. Preliminary experiments using agarose molded directly by 3D prints also showed negative effects on the meiotic progression of mouse oocytes, potentially due to leaching of ovotoxic compounds into the agarose [73]. Thus, we used a 3D-printed master mold to generate a safe, non-toxic silicone mold that could, in turn, be used to cast the agarose molds (Figure 1E). This material is flexible, does not leach compounds, and can be autoclaved. To assure no leachates were transferred to the agarose, a positive silicone cast was first made from the 3D print, followed by a final second negative cast. This technique has the added benefit that multiple silicone molds can be made from one 3D print, making the technique even more affordable. The final silicone molds reproducibly created the intended agarose culture molds that fit in individual wells of a 24-well plate as intended (Figure 1F).

In summary, we established a practical and economical method to design custom culture systems informed by biology and generated biocompatible hydrogel molds in a reproducible fashion without the risk of cytotoxicity. These molds can be adapted for other cultures, but this design is specific for mouse folliculogenesis.

### 3.2. Scaffold-Free Follicle Culture in Agarose Micromolds Supports Comparable Follicle Growth to Alginate Encapsulation

**Figure 2:**
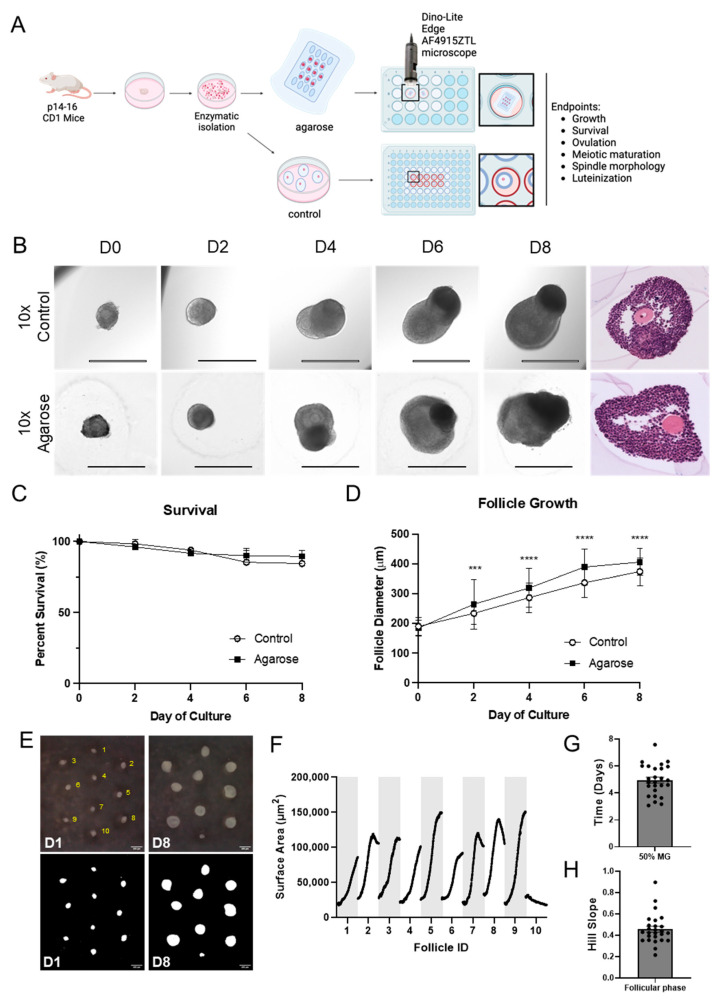
Follicles grown in agarose micromolds show similar survival and increased growth compared to controls. (**A**) Follicles were isolated from ovaries using enzymatic digestion and encapsulated in alginate (eIVFG) or seeded in agarose micromolds for culture. They were cultured for an 8-day follicular phase prior to ovulation and ex vivo luteinization. (**B**) Representative brightfield and H&E images of follicles across 8-day culture period for control follicles cultured in 0.5% alginate (**top**) and follicles cultured in agarose (**bottom**) at 10× magnification. (**C**) Quantification of follicle survival showed no significant differences between follicles cultured in alginate (control) or agarose. *n* = 4 replicates, 20 follicles each. (**D**) Quantification of follicle growth over an 8-day culture period indicates follicles cultured in agarose were significantly larger than controls by Day 2, with significantly increased growth sustained through Day 8. *** P-adj < 0.001; **** P-adj < 0.0001. *n* = 4 replicates, 20 follicles each. (**E**) Representative timelapse images of the beginning and end of the culture period. Follicles were numbered and thresholding allowed automated measurement of surface area. Scale bars: 400 µm. (**F**) Plot of surface area for each individual follicles during follicular phase. (**G**) Plot of time to half of maximal surface area and (**H**) hill slope. MG: maximal growth. *n* = 3 replicates, 10 follicles each (only surviving follicles quantified).

To verify that the agarose micromolds were biocompatible and able to support culturing of multilayer secondary follicles to the antral stage, we compared follicle morphology, survival, and growth from scaffold-free conditions to follicles grown in 0.5% alginate hydrogels (Figure 2A). Follicles grown in agarose micromolds maintained their 3D structure throughout the duration of culture and were morphologically similar to control follicles cultured in alginate hydrogels (Figure 2B). These follicles formed a fluid-filled antral cavity and contained mural granulosa cells and cumulus cells surrounding the oocyte (Figure 2B). Follicles grown In the agarose micromolds exhibited high survival through day 8 of culture, which was similar to follicles cultured in alginate hydrogels (89% ± 2% and 84% ± 5%, respectively, P-adj = 0.211) (Figure 2C). Interestingly, despite starting at similar initial diameters (190.3 ± 2.4 μm for control, 184.7 ± 2.4 μm for agarose micromolds, P-adj = 0.096), follicles cultured in the agarose micromolds reached significantly larger sizes by day 2 (P-adj < 0.0001) relative to control follicles cultured in hydrogels, and this increased growth was sustained through day 8 (P-adj < 0.0001; Figure 2D). The average terminal follicle diameter was 406.1 ± 4.1 μm in follicles cultured in the agarose micromold culture compared to 374.0 ± 4.1 μm in the control follicles (Figure 2D). These results indicate that agarose micromolds support follicle growth, resulting in a larger terminal diameter than standard methods.

An advantageous feature of the micromolds is that all follicles can be imaged simultaneously using timelapse imaging, since they are on the same plane (Figure 2E). We visualized the continuous growth across the follicular growth, ovulation, and luteinization phases (Supplemental Videos S1–S3). During the follicular phase, images were taken every 30 min for a total of 8 days. During analysis, every follicle was assigned a follicle identifier number for individualized tracking (Figure 2E and Figure S2). Using ImageJ analysis, we tracked and automatically calculated the follicles’ surface area (Figure 2F). The timelapse setup did not impact follicle viability, and the growing follicles had an average initial surface area of 23,806 ± 5556.2 μm^2^ and reached a terminal surface area of 115,751 ± 18,157.4 μm^2^. This corresponds to an idealized average diameter (assuming a perfect circle) of 172.37 ± 19.93 μm and 384.53 ± 32.49 μm, respectively (Figure 2F). During culture, follicle shapes vary but are generally oblong, and the measured surface area can differ depending on their orientation. Measuring this automatically and calculating the diameter of an idealized sphere can explain the differences in mean diameter and standard deviation compared to standard analysis that manually measures diameters.

Timelapse analysis is also able to demonstrate differences in growth dynamics, and morphokinetic information can be extracted for individual follicles. Similarly to analysis performed to measure dose–response [74], we can extract the time needed to reach half of the maximal surface area and hill slope of each growth curve. In other words, this gives an approximation of when growth acceleration is the highest and how steep the growth curve is, with the higher the hill slope the faster the growth. Growth acceleration was the highest at 4.96 ± 1.13 days, with a slope of 0.4571 ± 0.1411 (Figure 2G,H). Interestingly, some follicles (for example, ID 2, 7, and 8) reached their terminal size before others, followed by slight compaction before the completion of the follicular phase (Figure S2). These follicles also had the fastest times to reach 50% of maximal size and had the steepest slopes (0.6571, 0.8973, and 0.7219 for ID 2, 7, and 8, respectively), demonstrating that these data points can reflect variations in growth curves (Figure 2F–H and Figure S2). These differences may be due to follicle-intrinsic factors or a result of paracrine factors driving differential growth rates within individual follicles. Importantly, these metrics could be used to decode the optimal point during follicle growth for ovulation induction to yield the highest quality oocytes [75]. Taken together, we found that the agarose micromold enables timelapse imaging and analysis of follicle growth at a high temporal resolution.

### 3.3. Follicles Grown in Agarose Micromolds Ovulate COCs in a Consistent Spatial Orientation

**Figure 3:**
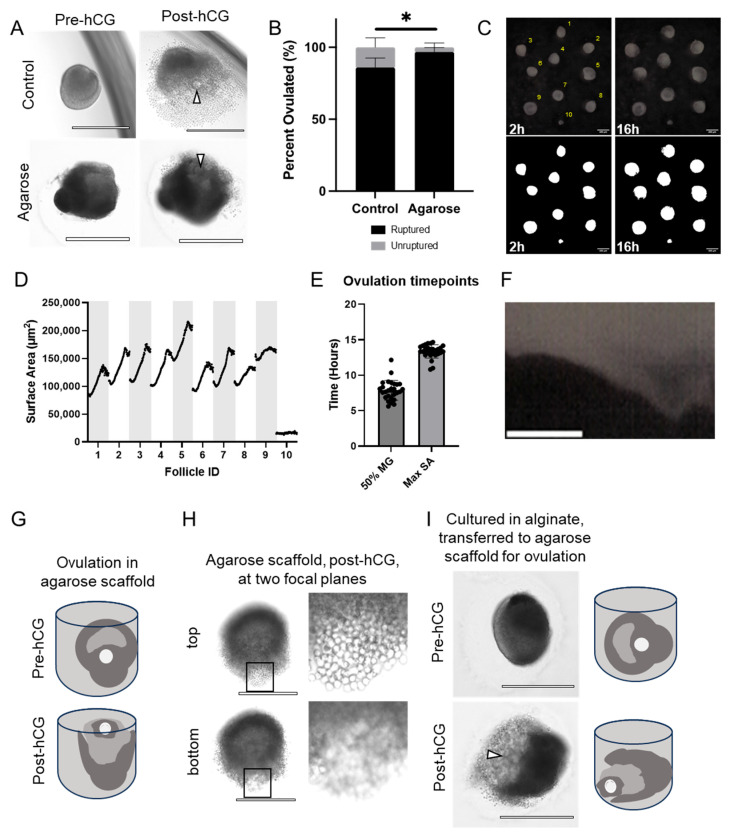
Follicles cultured and ovulated in agarose micromolds show spatial polarity during rupture. (**A**) Representative images of control (top) or agarose (bottom) follicles pre- and post-hCG. White arrowhead points to oocyte within COC. (**B**) Quantification of ovulation of follicles cultured in alginate or agarose with significantly higher ovulation rates in the agarose micromolds. * P-adj < 0.05. *n* = 3 replicates, 17–20 follicles each (follicles that did not survive to day 8 were excluded). (**C**) Representative timelapse images of beginning and end of the ovulatory period. Follicles were numbered and thresholding allowed measurement of surface area. Scale bars: 400 µm. (**D**) Plot of all surface area measurements for each individual follicle during ovulation. (**E**) Ovulation phase analysis with example fit of sigmoidal variable slope curves of follicle 3 during ovulation. Plot of time to half max growth and time at maximal surface area were plotted for the growing follicles. MG: maximal growth, SA: surface area. *n* = 3 replicates, 10 follicles each (only surviving follicles quantified). (**F**) Representative kymograph for follicle 3. Scale bar: 200 µm. (**G**) Schematic of follicle orientation within agarose microwells during ovulation. (**H**) Cumulus cells are visible at the top of the microwells, suggesting that cumulus–oocyte complexes are released in an upward direction during ovulation in follicles cultured in agarose, which was not observed in controls. (**I**) Representative images and schematic of follicles that were cultured in control conditions, removed from alginate, and then placed in agarose microwells for ovulation induction. White arrowhead points to oocyte within COC.

To further test the quality of follicles cultured in agarose micromolds, we assessed ovulation competency compared to control follicles. When induced to ovulate, follicles grown in agarose micromolds exhibited follicle rupture with release of an expanded COC (Figure 3A). However, the percentage of ruptured follicles was higher in follicles grown in the agarose micromolds compared to alginate, consistent with their larger terminal diameter (97% ± 2% compared to 82% ± 4%, P-adj = 0.031) (Figure 3B). For timelapse analysis, images were taken every 10 min over 14–16 h, and surface area was calculated automatically (Figure 3C, Supplemental Figure S3). The size of all follicles peaked and then decreased at the end of the ovulatory period (Figure 3D). We measured an average initial surface area of 110,111.56 ± 21,706.00 μm^2^, a peak of 164,636.67 ± 23,291.38 μm^2^, and a terminal surface area of 157,363.61 ± 23,914.63 μm^2^ (372.74 ± 35.55 μm, 456.74 ± 31.80 μm and 446.36 ± 33.57 μm normalized diameter, respectively) (Figure 3D). Growth kinetics were very similar across follicles, with the time to 50% maximal surface area occurring, on average, at 7.896 ± 1.397 h and an average slope of 0.2206 ± 0.08621 (Figure 3E). Of note, this is close to the average time of polar body extrusion (8.89  ±  0.08 h), marking the transition to metaphase of meiosis II, in our previous studies of oocyte maturation morphokinetic parameters [76]. During ovulation, the maximal surface area was measured at 13.36 ± 0.9360 h (Figure 3E). Supplementary Video S2 shows the continued growth and ovulation of the follicles. This expansion and contraction, which may be the result of documented ovulatory smooth muscle-like contractions [77,78,79,80], is also clearly visible when plotted as a kymograph (Figure 3F and Figure S4). This method can visualize the spatial position of the follicular wall along a selected line throughout the timelapse period. This is seen here as a thinning protrusion that peaks at the time of maximal surface area and then retracts.

Follicles grown in agarose micromolds exhibited a unique and consistent phenotype, whereby rupture occurred upwards towards the top of the microwells (Figure 3G). This is shown by the presence of cumulus cells, characterized by their distinct round appearance, in a higher focal plane than the rest of the follicle (Figure 3H, Supplemental Video S2). This may be more representative of in vivo physiology, where follicles rupture at the external facing side of the follicle wall, leading to release of the COC out of the ovary. Importantly, this phenotype does not appear to be due to the geometry of the microwells. When follicles that had been cultured in alginate hydrogels were removed from alginate, placed in the agarose microwells, and induced to ovulate, they ruptured consistently to the side rather than upwards (Figure 3I). Overall, these results suggest that follicles cultured in the agarose mold are capable of ovulating and recapitulate important features of in vivo ovulation.

### 3.4. Eggs Collected from Follicles Grown in Agarose Micromolds Are Meiotically Competent and Exhibit Normal Spindle Morphology

**Figure 4:**
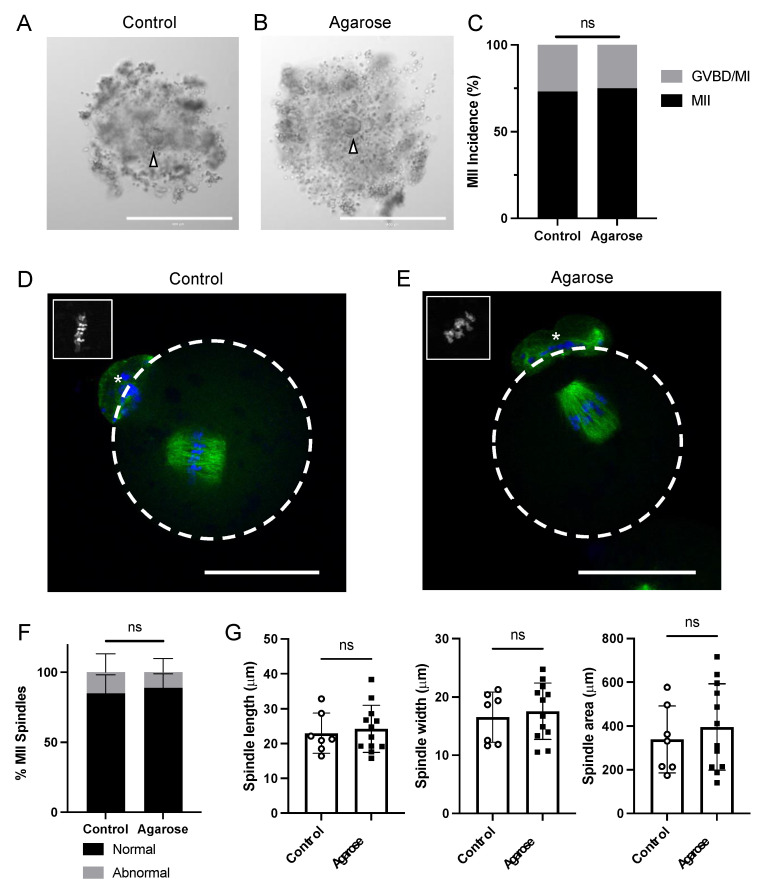
Oocytes collected from follicles grown in agarose micromolds are meiotically competent and exhibit normal spindle morphology. (**A**) Representative cumulus–oocyte complexes from post-ovulatory follicles grown in alginate or (**B**) in agarose. White arrowheads point to the oocyte. (**C**) Quantification of MII incidence in control- and agarose-cultured oocytes show no significant differences. *n* = 3 replicates, 13–15 oocytes each. (**D**) Representative meiotic spindles of oocytes from follicles grown in alginate or (**E**) in agarose micromolds. Dashed white line denotes oocyte outline, asterisk denotes polar body. Inset on top left shows chromosomes. Scale bar is 100 μm. (**F**) Quantification of spindle abnormalities in MII oocytes from follicles cultured in alginate and agarose micromolds shows no significant differences. *n* = 3 replicates, 6–8 oocytes each. (**G**) Quantification of spindle length (**left**), width (**middle**), and area (**right**) show no significant differences between oocytes collected from follicles grown in alginate or agarose. *n* = 7–12 oocytes. ns = not significant.

To determine whether follicles grown within agarose micromolds produced meiotically competent eggs, we collected gametes after ovulation and assessed meiotic stage and metaphase of meiosis II (MII) spindle parameters. Follicles cultured in both control conditions and agarose molds produced expanded COCs (Figure 4A,B). There were no significant differences in the percentage of cells that reached MII between control follicles and follicles grown in agarose (73% compared to 75%, P-adj > 0.999) (Figure 4C). Spindle morphology appeared similar between oocytes collected from control follicles and those grown in agarose (Figure 4D,E). There were no significant differences in the percentage of oocytes with abnormal spindle alignment between oocytes from control follicles and follicles grown in agarose (83.33% compared to 85.71%, P-adj > 0.999) (Figure 4F). We then quantified spindle length, width, and area (Figure 4G). There were no differences between spindle length (23.0 ± 5.7 μm in control, 24.2 ± 6.8 μm in agarose micromolds, P-adj = 0.682), spindle width (16.55 ± 4.5 μm in control, 17.5 ± 4.8 μm in agarose micromold, P-adj = 0.660), and spindle area (338.8 ± 153.2 μm in control, 395.8 ± 197.2 μm in agarose, P-adj = 0.521) (Figure 4G). Together, these results indicated that there were no significant differences in MII incidence or spindle size between follicles cultured in agarose micromolds and those cultured under control conditions.

### 3.5. Agarose Micromolds Sustain Hormonally Active Corpora Lutea following Ex Vivo Ovulation

**Figure 5:**
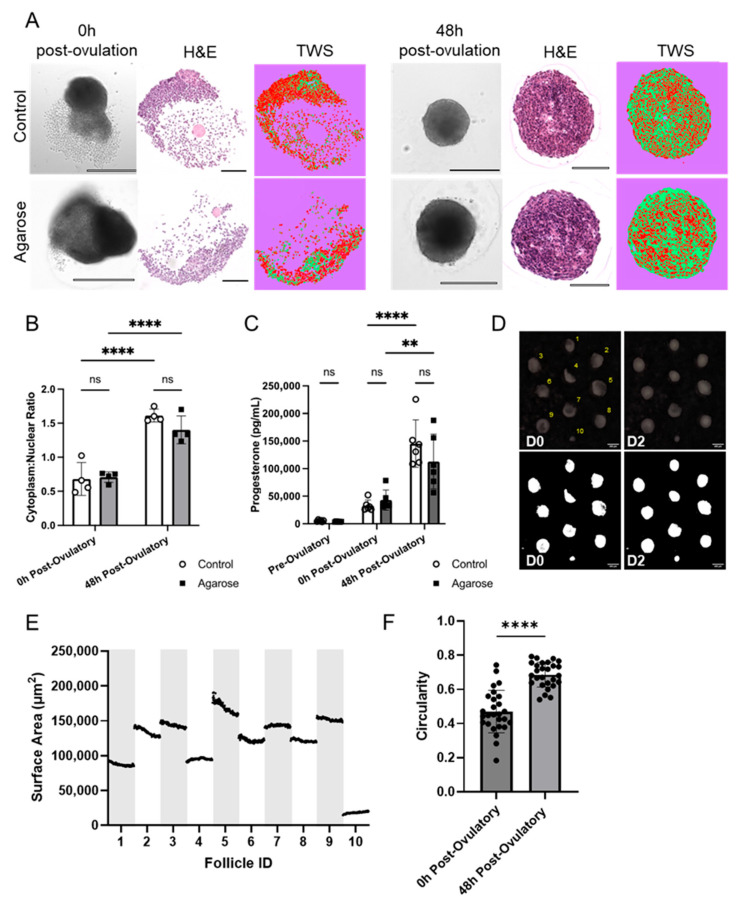
Formation of ex vivo corpora lutea is sustained in agarose micromold. (**A**) Representative brightfield and H&E images of follicles and ex vivo corpora lutea grown in alginate (**top**) and agarose micromolds (**bottom**). H&E-stained sections were analyzed with trainable weka segmentation (TWS) to separate nuclei (red), cytoplasm (green), and background (purple). (**B**) Cytoplasmic-to-nuclear ratio increases 48 h post-ovulation and is not significantly different in with CLs formed in agarose micromolds or alginate controls. ns: not significant. *n* = 4. (**C**) Progesterone levels are detectable, indicating functionality of ex vivo corpora lutea, and are not significantly different between those from follicles grown in agarose micromolds or alginate. ns: not significant. *n* = 4 replicates, each with media pooled between 10 follicles. (**D**) Representative images of beginning and end of the luteal period. Thresholding allowed measurement of surface area for every follicle. Scale bars: 400 µm. (**E**) Plot of all surface area measurements for each individual follicle during luteinization. (**F**) Plot of circularity measurements (*n* = 9 follicles) of ex vivo corpora lutea immediately after ovulation and 48 h later. *n* = 3 replicates, 10 follicles each (only surviving follicles quantified). For all statistical comparisons, ns = not significant; ** P-adj < 0.01; **** P-adj < 0.0001.

Following ovulation, the remnants of the follicle wall involute and undergo differentiation into a progesterone-producing corpus luteum. Ex vivo, this process is characterized by formation of a solid cellular structure, cellular hypertrophy as lipid deposits accumulate, and an increase in progesterone production over the course of 48 h following follicle rupture [81]. In our study, follicles cultured in agarose micromolds formed ex vivo corpora lutea that appeared morphologically similar to control follicles, both under brightfield imaging and in histologic sections (Figure 5A). We then quantified cellular hypertrophy by calculating the cytoplasmic-to-nuclear ratio in histologic sections and found that ex vivo corpora lutea generated within the micromolds exhibited a significant increase in cellular hypertrophy between the pre-ovulatory ratio and that measured 48 h after follicle rupture (P-adj = 0.016; Figure 5B). The degree of terminal cellular hypertrophy was also not significantly different between culture conditions (P-adj = 0.097; Figure 5B). Similarly, both culture conditions exhibited a similar, significant increase in progesterone production during the post-ovulatory period (Figure 5C). There was no significant difference between the terminal amount of progesterone produced by ex vivo corpora lutea from either condition at 48 h post-follicle rupture (145,286.5 ± 42,935.6 pg/mL in control, 112,827 ± 49,555.7 pg/mL in agarose micromold, P-adj = 0.365) (Figure 5C). During the luteal phase, we observed the remaining fragments regress into the ex vivo corpora lutea via timelapse imaging (Figure 5D,E). There was a significant increase in circularity during the luteal phase, with a calculated mean of 0.4702 ± 0.1241 directly after follicle rupture and 0.6865 ± 0.07313 48 h post-follicle rupture (Figure 5F; P-adj < 0.0001, *n* = 3, 27 follicles). On average, the first time the difference in circularity was significantly different from 0 h was at 26 h of culture (P-adj = 0.0276). This suggests that the agarose micromold is capable of sustaining formation of hormonally active ex vivo corpora lutea that are indistinguishable from those that form under standard culture conditions.

## 4. Discussion

The ability to isolate and culture follicles has been instrumental in the study of ovarian biology and has had direct clinical relevance for fertility preservation, toxicology studies, and drug screening [22,23,24,25,26]. Although multiple ex vivo follicle culture strategies have been developed over the past several decades, all have some limitations in terms of follicle and oocyte quality, throughput, or ease of adoption by new research groups [26,34,35,36,37,38,39,40,41,42,43,44,45,46,47,48,49,50,51,52,53,54,55,56,57,58,82,83]. To improve upon these challenges, we developed a novel custom agarose mold through a 3D printing method designed to support scaffold-free ex vivo follicle culture, survival, and growth. Though our study is not the first to demonstrate successful scaffold-free ex vivo follicle culture, it is, to our knowledge, the first to use a custom biocompatible micromold that is technically simple to use for culture, models novel in vivo-like features of ovulation, yields high-quality gametes, and leverages timelapse imaging and analysis to study morphologic changes during folliculogenesis and ovulation at a high temporal-resolution.

In this study, we harnessed the advantages of 3D SLA printing, namely printing resolution, customizability, and speed of iteration, while mitigating a key drawback, the release of cytotoxic leachates during culture [71,73]. We chose to use SLA as a 3D-printing technique rather than directly using agarose bioprinting due to its high printing resolution, and the ability for our design to be more widely accessible for other research groups. To avoid issues with cytotoxicity, we created silicon molds for agarose micropatterning, which also made the method more economical as many silicone molds can be created from one 3D print. We used two silicon casts to minimize contact of the agarose to the initial 3D printed mold and, therefore, minimize risk of cytotoxicity. The specialized microwell array was designed to accommodate the significant size increase of murine multilayer secondary follicles during culture and ovulation. Overall, the agarose micromolds sustained follicle growth and function throughout the follicular, ovulatory, and luteal periods. Follicular survival and overall morphology, meiotic progression, spindle measurements in oocytes, and ex vivo corpus luteum formation were consistent or improved compared to follicles cultured in 0.5% alginate. The process of oocyte meiosis is highly susceptible to errors and perturbations and would result in reduced meiotic resumption, abnormal spindles, or abnormal chromosome alignment [76]. Our results confirm that there are no differences in MII incidence, spindle abnormalities, or spindle size in the agarose molds compared to alginate-based culture, which provides strong evidence of biocompatibility of our new method. 

To determine the efficacy of our agarose micromolds for follicle culture, we made several comparisons to eIVFG, the current gold standard [34,54]. Multilayer secondary follicles grown in our agarose microwells had improved survival and growth compared to follicles cultured in alginate. Additionally, after ex vivo ovulation, there was a significant increase in rupture rates for follicles cultured in agarose compared to alginate. This could be, in part, because the follicles could be ovulated directly in the micromolds and did not need to be moved or released from encapsulation. Intriguingly, follicles cultured in agarose consistently had a spatial orientation of ovulation, with COCs being extruded upwards. Importantly, this polarity was not present when follicles cultured in alginate were induced to ovulate within the micromolds. This suggests that the spatial orientation of the antrum and follicle rupture is encoded early in folliculogenesis, which allows follicle rupture to occur in the direction of the most permissive biomechanical environment to facilitate COC release. This may better recapitulate spatial programming that follicles undergo in vivo, as release of the COC towards the exterior of the ovary is essential for fertilization capacity [12,77,84,85,86]. The signaling mechanisms that drive this polarity and the orientation of follicle rupture are active areas of investigation.

The utility of custom micromolds for scaffold-free cultures has been widely demonstrated in organoid models [63,87,88,89]. While our technique is not the first scaffold-free follicle culture strategy, we were able to integrate many of these advantages to achieve improvements over existing scaffold-free and encapsulated techniques [26,34,35,36,37,38,44,45,46,47,48,49,50,51,52,53,54,55,56,57,58,82,83]. Firstly, eliminating the need for encapsulation to maintain 3D architecture makes it less technically demanding and more reproducible, which could eventually lead to incorporation in other translational and clinical settings, such as with drug screening, fertility preservation, and species conservation. Secondly, the overall structure of our microwells protects the follicles during growth to prevent migration and merging with adjacent follicles while remaining in a shared culture environment. This increases ease and efficiency of culture media changes and allows modulation of follicle number and stage, more like in vivo physiology, to determine the impact of paracrine factors from group culture. Thirdly, the ability to culture multiple follicles within the same focal plane facilitated faster, automated imaging of follicles throughout culture. Typically, multilayer secondary follicles are individually encapsulated with one follicle per well of a 96-well plate and require significant time for imaging as they may not be within the same plane of focus.

Another major advance provided by this technique is the ability to perform continuous timelapse imaging, which allows for automatic and individualized quantification of the surface area of follicles throughout the different follicular, ovulatory, and luteal growth phases and dynamic information on growth kinetics and morphology. In the present study, we described three different quantitative metrics that can be derived from timelapse imaging throughout culturing. The first two, the timepoint when fifty percent of the maximal surface area is reached and the maximal steepness of the growth curve, can differentiate variations in growth rate of individual follicles during culture and provide insights into changes to follicle morphology during ovulation. This could be particularly useful for future studies that incorporate follicles of different stages that may have different growth velocities, selection of follicles to ovulate at the optimum point in their growth for maximum oocyte quality, or treatment of follicles with drugs that may alter folliculogenesis or ovulation [58]. Future studies will assess if the timing of plateaued growth or 50% of maximum growth can be used to optimize ovulation timing and improve oocyte quality and fertilization capacity [75]. The third metric we derived was circularity, which showed a significant increase 48 h post-ovulation. This metric may help track the luteinization capacity of individual follicles in the post-ovulatory period.

Although our culture system improves upon many of the challenges that persist with existing follicle culture methods, there are still some areas of further improvement. Biomechanical support is necessary for growth of follicles, particularly at early stages of development [34,57,90,91,92]. Future studies will be needed to determine whether the current mold design provides sufficient biomechanical support for the growth of primary or early secondary follicles. Further studies on follicles of different stages may require alterations to the mold design or use of different biomaterials with different mechanical properties. Additionally, it would be informative to complete analysis of expression of key genes enriched during folliculogenesis, ovulation, and luteinization to confirm that follicles are indeed recapitulating in vivo molecular signatures. Finally, our use of an inert biomaterial does not provide follicles with biomimetic substrates that have been shown to support follicle growth. In future studies, we may be able to generate micromolds with more complex biomaterials [1,86,90,93,94,95,96,97,98,99,100,101,102,103,104,105,106].

The adaptability of this system will be crucial for several future research applications. The current microwell design can be easily adapted, for example to co-culture follicles of different developmental stages or to accommodate the different growth patterns in larger species, which will need specific dimensions and proximity. This could potentially be applied to ex vivo culture of human follicles, which have only been shown to yield mature oocytes in a two-step system that incorporates scaffold-free culture in ultra-low attachment plates [40]. Our culture system, which can be adapted to the specific geometry of human antral follicles, may be capable of generating mature human gametes from preantral follicles. This would serve as a groundbreaking tool for fertility preservation as mature gametes could potentially be cultured from surgically isolated ovarian tissue from patients undergoing cancer treatment or with fertility-threatening diagnoses [27,28,29,30,31]. This assay could similarly be used for fertility preservation in other mammalian species, many of which have had limited success in ex vivo culture with existing techniques [107,108,109]. Mature gametes could be derived and then fertilized or frozen to generate a biobank for species conservation. Additionally, the agarose mold is compatible with the LATTICE platform, a microphysiological system (MPS) that represents a powerful next step in advancing ex vivo models [59,110]. Microfluidic technologies have been applied in the culture of individual follicles [111,112,113], as well as explants [59,113]. We can use this device to integrate follicles with other tissues to model complex reproductive physiology and perform more biomimetic cultures. Beyond this, leveraging timelapse analysis could convert this platform into an ideal biological screening tool. Advanced analysis of morphology and growth kinetics could be advanced based on the use of neural networks, machine learning, and artificial intelligence, as has been done for organoids and embryos [114,115,116,117,118]. These advanced computational techniques could be applied to our model to study the effects of toxic chemicals or environmental pollutants on follicle maturation, ovulation, and hormone production at a high temporal resolution [22,23,24,25,26,27,28,29]. Additionally, the same techniques could be used in our assay to test potential contraceptive agents that block follicle rupture without impacting the endocrine axis [58]. Compounds with potential contraceptive efficacy could be tested on multiple follicles within a single well to assess the impact on follicle rupture, oocyte maturation, and hormone production during the luteal period. The ability to treat and monitor follicles simultaneously would increase the throughput of this complex phenotypic assay. 

## 5. Conclusions

The scaffold-free culture method described here supports growth and development of murine multilayer secondary follicles, presenting similar survival and development rates to the current gold standard, eIVFG. Moreover, ovulation rates are improved, follicles produce meiotically competent oocytes, and the molds sustain the formation of hormonally active ex vivo corpora lutea. We also observed a distinct apical ovulation phenotype that may better recapitulate spatial polarization seen in vivo during the periovulatory period [85]. The scaffold-free agarose micromold culture system could, therefore, be an ideal method to study the role of biomechanical force on driving spatial polarity in the follicle prior to the ovulatory stimulus. The microwells were specifically designed to allow follicles to go through the follicular growth, ovulation, and luteinization phases in the same focal plane, allowing for continuous monitoring via timelapse imaging, as well as facilitating downstream processing, such as paraffin embedding and sectioning. The customizable agarose mold is a powerful potential tool for studying basic biology, toxicology, drug screening, and novel contraceptive development, and for the development of fertility preservation strategies.

## Data Availability

The datasets analyzed during the current study are available from the corresponding author on reasonable request.

## Supplementary Materials

The following supporting information can be downloaded at: https://www.mdpi.com/article/10.3390/bioengineering11070719/s1, Figure S1: Print orientation affects final microwell geometry; Figure S2: Growth curve analysis during follicular phase; Figure S3: Growth curve analysis during ovulation; Figure S4: Kymograph visualization of ovulation in agarose micromolds; Video S1: Timelapse of 8-day follicular phase; Video S2: Timelapse of 16-h in-vitro maturation phase; Video S3: Timelapse of 2-day Luteal phase.
