# Supplementary material for: Three-Dimensionally Printed Agarose Micromold Supports Scaffold-Free Mouse Ex Vivo Follicle Growth, Ovulation, and Luteinization
Source: Bioengineering (Basel). 2024 Jul 15;11(7):719. doi: 10.3390/bioengineering11070719 (PMC11274170; doi:10.3390/bioengineering11070719)
Supplement: Supplementary file 1 [file bioengineering-11-00719-s001.zip › Final_bioengineering-3071002-supplementary.pdf]

*Supplementary Materials*

# Three-Dimensionally Printed Agarose Micromold Supports Scaffold-Free Mouse Ex Vivo Follicle Growth, Ovulation, and Luteinization

**Emily J. Zaniker [1,†], Prianka H. Hashim [1,†], Samuel Gauthier [1], James A. Ankrum [2], Hannes Campo [1,*] and Francesca E. Duncan [1,*]**

[1] Department of Obstetrics and Gynecology, Feinberg School of Medicine, Northwestern University, Chicago, IL 60611, USA; emily.zaniker@northwestern.edu (E.J.Z.); prianka.hashim@northwestern.edu (P.H.H.); samuelj.gauthier@gmail.com (S.G.)
[2] Roy J. Carver Department of Biomedical Engineering, Pappajohn Biomedical Institute, University of Iowa, Iowa City, IA 52245, USA; james-ankrum@uiowa.edu
* Correspondence: hannes.campo@northwestern.edu (H.C.); f-duncan@northwestern.edu (F.E.D.); Tel.: +1-312-503-1541 (H.C.); +1-312-503-1084 (F.E.D.)
† These authors contributed equally to this work.

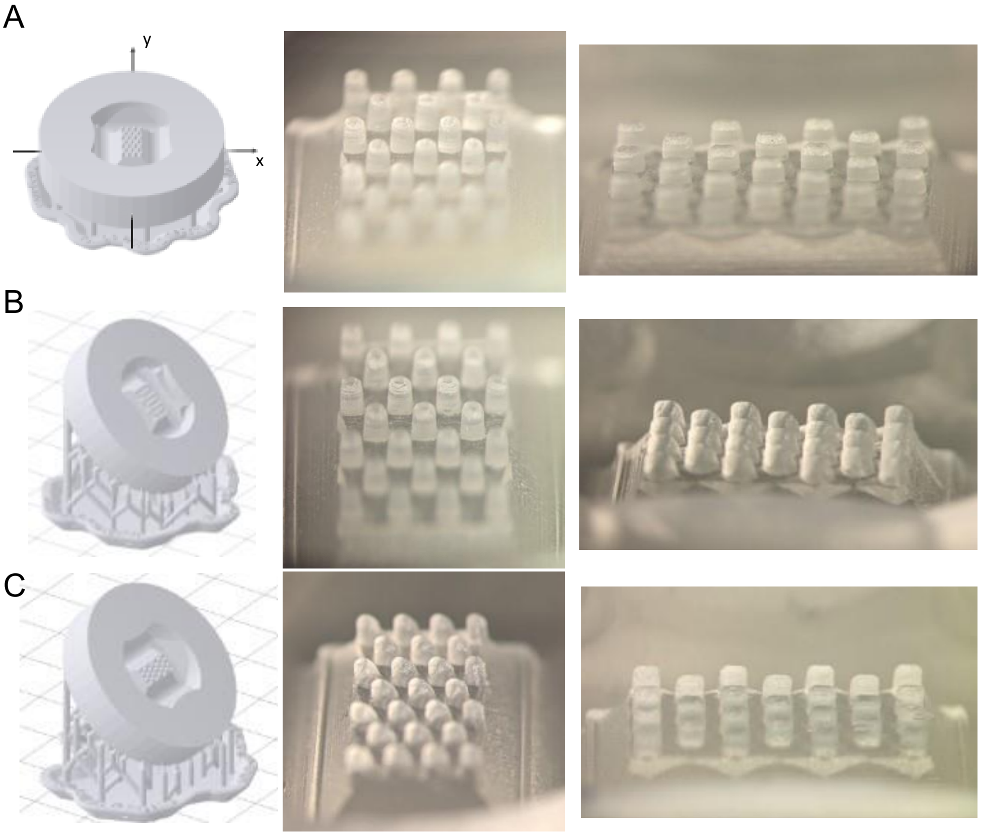


**Figure S1.** Print orientation affects final microwell geometry. **(A)** Flat print orientation results in correct print resolution. (**B**) 45-degree tilt along the x axis leads to well distortions in the longitudinal direction (right). (**C**) 45-degree tilt along the y axis leads to well distortions in the transversal direction (left).

A

B

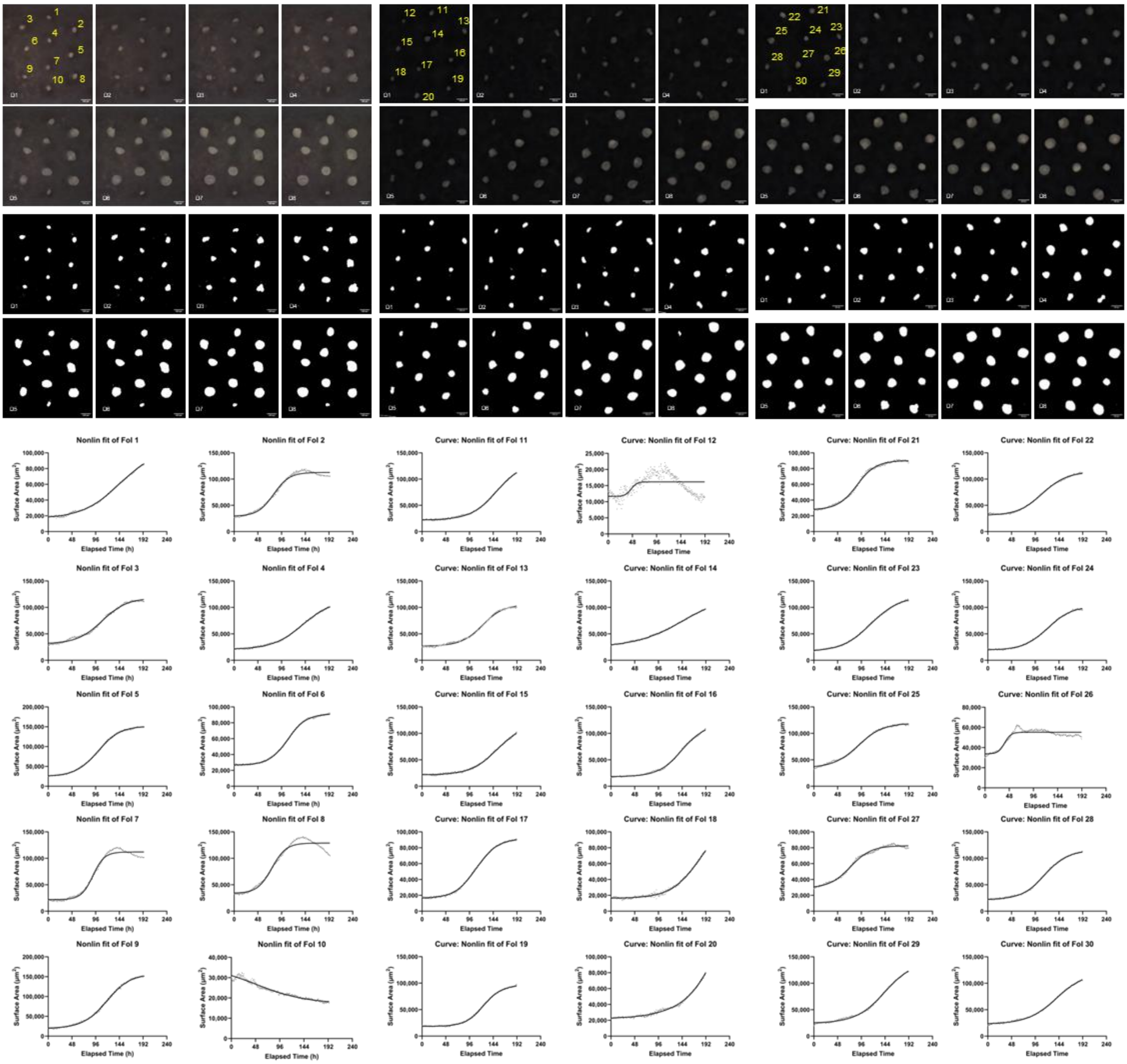

C

| Sigmoidal dose-response (variable slope) – Best fit values | | | | | | | | | | |
|---|---|---|---|---|---|---|---|---|---|---|
| | Follicle 1 | Follicle 2 | Follicle 3 | Follicle 4 | Follicle 5 | Follicle 6 | Follicle 7 | Follicle 8 | Follicle 9 | Follicle 10* |
| Bottom | 16,688 | 29,381 | 31,241 | 20,843 | 24,933 | 26,730 | 22,085 | 33,989 | 19,634 | 17,143 |
| Top | 104,633 | 112,788 | 118,095 | 114,531 | 152,006 | 91,401 | 112,083 | 128,970 | 158,145 | 36,367 |
| LogEC50 | 6.010 | 3.324 | 4.546 | 5.797 | 4.192 | 4.518 | 3.760 | 3.173 | 4.941 | 2.035 |
| HillSlope | 0.2755 | 0.6571 | 0.4113 | 0.3418 | 0.4621 | 0.5523 | 0.8973 | 0.7219 | 0.4304 | -0.2062 |
| | Follicle 11 | Follicle 12* | Follicle 13 | Follicle 14 | Follicle 15 | Follicle 16 | Follicle 17 | Follicle 18 | Follicle 19 | Follicle 20* |
| Bottom | 22,106 | 16,175 | 27,037 | 24,914 | 21,530 | 18,347 | 16,144 | 16,329 | 18,306 | 21,675 |
| Top | 132,285 | 116,65 | 105,445 | 126,324 | 118,185 | 117,540 | 917,24 | 120,012 | 96,272 | Unstable |
| LogEC50 | 6.303 | 1.887 | 5.077 | 6.131 | 6.329 | 5.959 | 4.566 | 7.582 | 5.173 | 18.22 |
| HillSlope | 0.3937 | -1.767 | 0.4864 | 0.2150 | 0.3880 | 0.4422 | 0.4823 | 0.3516 | 0.5645 | 0.2133 |
| | Follicle 21 | Follicle 22 | Follicle 23 | Follicle 24 | Follicle 25 | Follicle 26* | Follicle 27 | Follicle 28 | Follicle 29 | Follicle 30 |
| Bottom | 27,780 | 30,807 | 16,726 | 19,296 | 35,195 | 33,734 | 29,152 | 22,155 | 24,641 | 22,959 |
| Top | 90,239 | 113,808 | 121,088 | 102,567 | 119,383 | 55,213 | 82,693 | 114,928 | 142,929 | 121,447 |
| LogEC50 | 3.628 | 4.596 | 4.801 | 5.048 | 3.747 | 1.611 | 3.071 | 4.787 | 5.991 | 5.807 |
| HillSlope | 0.5406 | 0.4211 | 0.3548 | 0.4249 | 0.4198 | 1.672 | 0.4926 | 0.4515 | 0.3553 | 0.3517 |

**Figure S2.** Growth curve analysis during follicular phase. (**A**) Representative images of microarray capture for each day of the 8-day follicular culture period (n = 3, 30 follicles). (**B**) Timelapse growth curves for all follicles were fitted using four parameter logistic regression model. (**C**) Growth curve parameters extracted from each individual follicle. Follicles were excluded if they were not viable, or if the plot yielded no results (red).

A

B

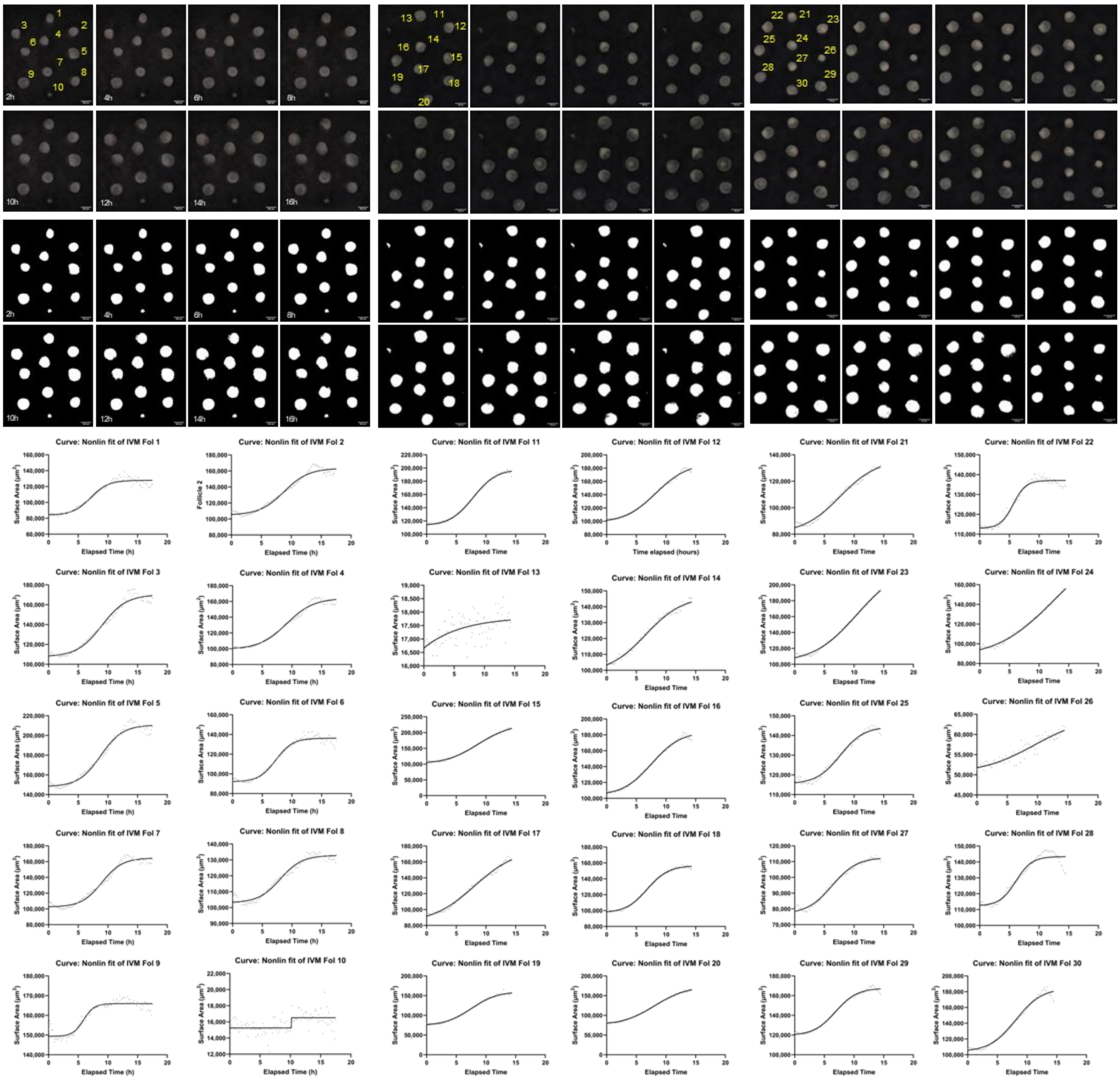


C

| Sigmoidal dose-response (variable slope) – Best fit values | | | | | | | | | | |
|---|---|---|---|---|---|---|---|---|---|---|
| | Follicle 1 | Follicle 2 | Follicle 3 | Follicle 4 | Follicle 5 | Follicle 6 | Follicle 7 | Follicle 8 | Follicle 9 | Follicle 10* |
| Bottom | 84,115 | 104,924 | 107,664 | 99,451 | 148,068 | 92,025 | 102,388 | 103,013 | 149,356 | 15,254 |
| Top | 127,900 | 163,401 | 170,131 | 164,097 | 210,704 | 136,272 | 164,677 | 132,884 | 165,963 | 16,518 |
| LogEC50 | 7.079 | 8.788 | 8.951 | 9.097 | 8.764 | 7.284 | 8.979 | 8.022 | 5.890 | 10.17 |
| HillSlope | 0.2927 | 0.2124 | 0.2096 | 0.1927 | 0.2339 | 0.3053 | 0.2527 | 0.2373 | 0.4842 | Unstable |
| | Follicle 11 | Follicle 12 | Follicle 13* | Follicle 14 | Follicle 15 | Follicle 16 | Follicle 17 | Follicle 18 | Follicle 19 | Follicle 20 |
| Bottom | 112,884 | 97,703 | -6,238,255 | 98,018 | 100,163 | 103,319 | 82,922 | 97,282 | 73,832 | 75,055 |
| Top | 198,393 | 187,501 | 17,822 | 146,753 | 226,605 | 184,276 | 176,685 | 156,546 | 160,877 | 173,979 |
| LogEC50 | 7.853 | 8.326 | -53.26 | 6.546 | 8.700 | 7.583 | 8.081 | 6.825 | 7.526 | 8.091 |
| HillSlope | 0.2226 | 0.1648 | 0.07006 | 0.1385 | 0.1608 | 0.1776 | 0.1220 | 0.2461 | 0.1983 | 0.1601 |
| | Follicle 21 | Follicle 22 | Follicle 23 | Follicle 24 | Follicle 25 | Follicle 26* | Follicle 27 | Follicle 28 | Follicle 29 | Follicle 30 |
| Bottom | 80,531 | 112,985 | 102,048 | 86,110 | 115,402 | 50,297 | 76,768 | 112,386 | 119,660 | 103,817 |
| Top | 136,507 | 137,083 | 221,451 | 198,409 | 144,491 | 64,342 | 112,734 | 143,409 | 167,726 | 184,640 |
| LogEC50 | 7.658 | 5.629 | 10.35 | 12.15 | 7.671 | 9.380 | 6.301 | 6.087 | 6.876 | 8.095 |
| HillSlope | 0.1390 | 0.4075 | 0.1217 | 0.09256 | 0.2179 | 0.1002 | 0.2065 | 0.3222 | 0.2393 | 0.1988 |

**Figure S3.** Growth curve analysis during ovulation. (**A**) Representative images of microarray capture for each day of the 8-day follicular culture period (n = 3, 30 follicles). (**B**) Timelapse growth curves for all follicles were fitted using four parameter logistic regression model. (**C**) Growth curve parameters extracted from each individual follicle. Follicles were excluded if they were not viable, or if the plot yielded no results (red).

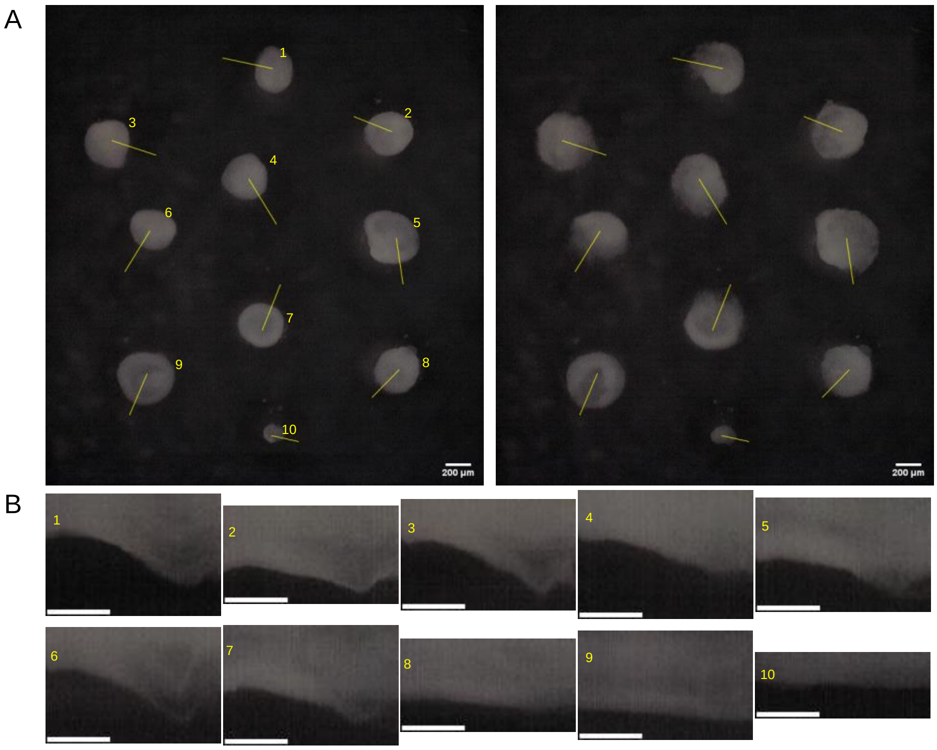


**Figure S4.** Kymograph visualization of ovulation in agarose micromolds. (**A**) For each follicle a line was drawn following the path where ovulation occurred most visibly. (**B**) Kymograph for each individual follicle made from 106 images during ovulation window.

Video S1. Timelapse of 8-day follicular phase. Images were taken every half hour, 30 fps. Scale bar 400 µm.

Video S2. Timelapse of 16-hour in-vitro maturation phase. Images were taken every 10 minutes, 30 fps. Scale bar 400 µm.

Video S3. Timelapse of 2-day Luteal phase. Images were taken every 10 minutes, 90 fps. Scale bar 400 µm.

**Disclaimer/Publisher's Note:** The statements, opinions and data contained in all publications are solely those of the individual author(s) and contributor(s) and not of MDPI and/or the editor(s). MDPI and/or the editor(s) disclaim responsibility for any injury to people or property resulting from any ideas, methods, instructions or products referred to in the content.
